# A Novel System for Physiological Signal Monitoring and Health-Informed Electrotactile Feedback for First Responders

**DOI:** 10.3390/s26072054

**Published:** 2026-03-25

**Authors:** Bojan Jorgovanović, Vojin Ilić, Nikola Jorgovanović, Marina Peña-Díaz, Goran Bijelić, Jovana Malešević, Miloš Kostić, Matija Štrbac

**Affiliations:** 1 Faculty of Technical Sciences, University of Novi Sad, 21000 Novi Sad, Serbia; vojin@uns.ac.rs; 2 Global Electronic Solutions doo, 21000 Novi Sad, Serbia; 3 Tecnalia, Basque Research and Technology Alliance (BRTA), 20009 Donostia-San Sebastian, Spain; marina.pena@tecnalia.com (M.P.-D.); goran.bijelic@tecnalia.com (G.B.); 4 Tecnalia Serbia Ltd., 11000 Belgrade, Serbia; jovana.malesevic@tecnalia.com (J.M.); milos.kostic@tecnalia.com (M.K.); matija.strbac@tecnalia.com (M.Š.)

**Keywords:** physiological signal acquisition, multimodal bio-sensing, electrotactile stimulation feedback, real-time monitoring, wearable biosensors, closed-loop biofeedback

## Abstract

**Highlights:**

**What are the main findings?**

- A multimodal wearable system with physiological and electrochemical acquisition modules and electrotactile stimulation which enables data-driven feedback for first responders via a remote command centre.
- Laboratory and field evaluations demonstrate accurate electrochemical sensing (including sodium-based dehydration assessment), reliable vital sign monitoring, and over 80% success in continuous data transmission and electrotactile feedback.

**What are the implications of the main findings?**

- The proposed system supports safer and more effective first responder operations through continuous health monitoring and timely, condition-based interventions.
- Its modular and robust design facilitates integration into operational environments and provides a foundation for advanced remote health supervision in high-risk missions.

**Abstract:**

Ensuring the safety and effectiveness of first responder teams during critical missions requires real-time health monitoring and responsive intervention systems. This study presents a novel system comprising a multimodal wearable device integrated with a remote command centre, designed to support the physiological monitoring and guidance of first responders in the field. The wearable device includes three main components: a physiological and biochemical signal acquisition unit, an electrotactile stimulation unit and a powerful communication interface. The acquisition unit continuously samples heart rate, body temperature, and biochemical markers from sweat, transmitting this data wirelessly to the remote command centre. The transmitted physiological data could be analyzed at the command centre and, based on the inferred first responder condition, appropriate feedback commands could be issued back to the corresponding wearer. The commands are then executed by the electrotactile stimulation unit on the wearable device. Initial testing in laboratory settings confirmed the system’s ability to generate accurate electrochemical readings and dehydration assessment through changes in bulk ionic conductivity. Electrochemical impedance spectroscopy showed good agreement with a commercial potentiostat. Heart rate and temperature readings demonstrated satisfying accuracy with minor removable artifacts. Field trials with first responders validated continuous signal transmission and electrotactile feedback with over 80% success. These results confirm the system’s robustness and modularity, supporting its application in operational environments.

## 1. Introduction

In recent decades, missions involving first responders in demanding environments, such as wildfire response or high-altitude search and rescue, have become increasingly common rather than exceptional. As this tendency is expected to continue, the threats to the health and safety of these personnel are also increasing. To reduce the likelihood of life-threatening injuries or long-term health impacts, it is crucial to track key physiological indicators of first responders in a way that provides prompt and meaningful feedback, without impairing their effectiveness in the field [1,2].

With the rapid development of wearable devices and IoT devices in general, as well as flexible and stretchable materials suitable for producing both devices and sensors, real-time monitoring of such parameters is becoming significantly more feasible and reliable. Numerous wearable devices used for monitoring physiological parameters have been developed. The most common types of devices found in the literature are wearable devices, whether attached to a piece of clothing [3,4,5], or equipped independently [6,7,8], although implantable devices are also opted for in certain situations [9]. The devices gather data using various types of sensors for monitoring physiological parameters such as ECG and/or heart rate (HR) [5,10,11,12], body temperature [6], blood pressure [6,13,14], SpO_2_ [11,12,15], sweat rate [7], etc.

The integration of vital signs monitoring with electrochemical measurements in a single wearable device offers a comprehensive approach to health monitoring, particularly for first responders operating in extreme environments. Combining physiological signals such as temperature and ECG/HR with electrochemical measurements for key biomarkers detection (e.g., ions, lactate, and cortisol) can provide a more holistic understanding of an individual’s health status. This multimodal sensing approach is an essential prerequisite for future health assessment, allowing for the early detection of conditions such as dehydration, stress, and fatigue, which are critical for maintaining operational efficiency and safety in high-risk situations. Recent advancements in multimodal sensing technologies have demonstrated significant potential in improving health monitoring accuracy and reliability. For instance, flexible and stretchable materials have enabled the development of wearable devices that can seamlessly integrate multiple sensor types without compromising comfort or mobility [16,17,18].

The fusion of electrophysiological and electrochemical data has been shown to enhance the detection of physiological anomalies by providing complementary information that single-modality sensors might miss [19,20]. This integrated approach facilitates a more detailed and timely understanding of the wearer’s condition, leading to better-informed decisions and interventions. Advanced techniques such as machine learning and signal processing have been instrumental in extracting meaningful insights from the aggregated data, thereby improving the accuracy of health assessments [21,22,23]. For instance, the integration of electrochemical biosensors into wearable, portable, and implantable devices has enabled real-time monitoring of various biomarkers with high sensitivity and specificity [24]. At the same time, multimodal electrophysiological sensing approach, coupled with advanced data fusion techniques, can provide a more holistic assessment of health status and improved diagnostic accuracy [25].

The development of wearable biosensors that can continuously monitor biochemical markers such as glucose, lactate, and cortisol alongside vital signs has therefore marked a significant advancement in personalized health monitoring. For example, sensors that measure sweat composition to track dehydration and electrolyte imbalance have been integrated with heart rate monitors to provide a comprehensive picture of a user’s hydration status and cardiovascular health [26]. In this regard, sweat is a vital bodily fluid that helps regulate temperature and maintain electrolyte balance. As the body loses water and electrolytes through sweating, changes in the ionic composition and electrical conductivity of sweat can provide valuable insights into an individual’s hydration status. Sweat conductivity reflects the total ionic strength of the fluid and is strongly influenced by the concentrations of major electrolytes, primarily sodium and chloride. Sodium is an essential electrolyte that plays a critical role in maintaining fluid balance, nerve function, and muscle contraction [27]. Sweat Na^+^ concentration can vary considerably among individuals and when the body is well-hydrated, the sodium concentration in sweat typically ranges from 10 to 90 mM [28]. However, as dehydration sets in, the sodium concentration in sweat can drop even further, indicating a critical need for rehydration. Conversely, excessive sodium loss through sweat can lead to hyponatremia, a condition characterized by low sodium levels in the blood. Furthermore, dehydration is often accompanied by changes in physiological parameters such as heart rate and body temperature, which can serve as additional indicators of hydration status and cardiovascular diseases [29]. For example, dehydration can cause an increase in heart rate as the body attempts to compensate for decreased blood volume, while body temperature can rise due to impaired sweat evaporation and heat dissipation [27]. Accurate measurement of changes in ionic conductivity in sweat, in conjunction with monitoring of heart rate and body temperature, can provide a comprehensive picture of an individual’s hydration status and help prevent dehydration-related complications. These combined measurements are crucial for first responders who need real-time data to make informed decisions during physically and mentally demanding situations.

However, gathering data is only a part of the problem and is useful to first responders only if the acquired information can be fed back to the user wearing the device. Providing adequate feedback to the user of the device can be achieved in several ways. The most obvious way of conveying information to a human is via visual [30] or audio cues [31]. However, a study has shown that the most effective means of providing feedback is via tactile stimulation [32], especially in stressful situations. Some ways of evoking tactile sensation include electrotactile stimulation [33,34,35] and vibrotactile stimulation [36,37].

This paper proposes a new system designed specifically for assisting first responders in their missions, enabling continuous monitoring of their physiological signals, through physical and electrochemical transduction, and providing electrotactile feedback about the health status of their organism as well as communicating with a remote command centre. This study further evaluates the application of the data acquisition system in laboratory conditions in measuring heart rate, temperature and dehydration state through bulk ionic conductivity in sweat. Furthermore, the entire concept, including overall system functionality and communication reliability, is also verified in a field trial conducted with actual first responders in a simulated action.

## 2. Materials and Methods

### 2.1. System Description

#### 2.1.1. System Topology

**Figure 1:**
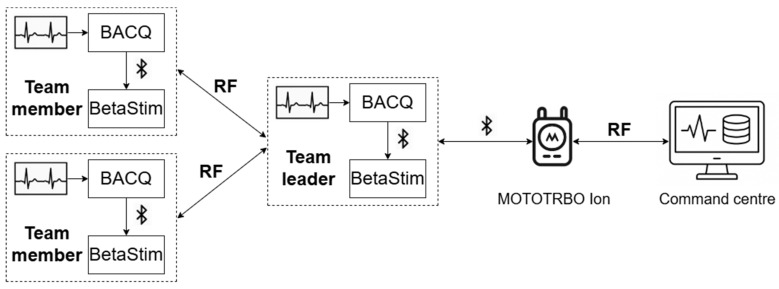
Topology of the entire system. The figure depicts a team consisting of two team members and a team leader, each equipped with a BACQ and BetaStim wearable device. Members’ BACQs transmit data to the leader’s BACQ over an RF link. The leader’s smart radio, connected to his/her BACQ via Bluetooth, forwards the collected data to a remote command centre. Additionally, feedback commands are sent to the leader’s smart radio and are distributed to the corresponding team members via the same communication channels.

First responder missions usually involve a team of first responders and a remote command centre. The team consists of several team members and a team leader who is the only member that has an open communication channel with the command centre [38]. The system, shown in Figure 1, was designed with this structure in mind and involves a specifically manufactured wearable system worn by each member of the first responder team (including the team leader). The system also utilizes the resources of the command centre for carrying out algorithmic processing. The link between the command centre and the first responder team, as shown in Figure 1, is established via radio frequency (RF) communication through a mobile radio device. The team leader is equipped with the MOTOTRBO Ion portable smart radio produced by Motorola (Chicago, IL, USA) which is connected to his/her wearable system via Bluetooth. The team members’ wearable systems are not able to access the mobile device directly but only through the team leader’s system. Because of this, an RF communication channel was established between the team leader’s and every team member’s wearable system. The RF communication channel provides reliable communication between the team leader and team members at a distance of about a hundred metres in real operating conditions. The wearable system is separated into two devices, the Beta Acquisition device (BACQ) and the BetaStim device, which are connected to each other via Bluetooth Low Energy. BACQ is configured as a GATT client and BetaStim as a GATT server. Both devices are powered by 3.7 V Li-poly rechargeable batteries and integrate electronic circuits for battery charging, battery protection and battery monitoring. Power consumption was considered in the design, and both devices are capable of operating for up to 12 h under intensive use on a fully charged battery. The reason why two separate devices were designed instead of one was twofold. Firstly, the devices could be placed closer to the connection points between the skin and electrodes and/or sensors, eliminating the need for long wires. Secondly, the overall device dimensions were significantly reduced compared to a single device that combines both functionalities.

It should be emphasized that the devices presented in this work are prototype devices intended to demonstrate feasibility and core functionality. The developed devices are designed in accordance with principles for biomedical wearable electronic devices, with some examples presented in [39,40,41]. Transitioning these prototypes into medical grade devices would require validation and certification. This includes, but is not limited to, comprehensive design validation, assessment of PCB integrity and reliability under expected operating conditions [42,43].

#### 2.1.2. Data Acquisition Device

**Figure 2:**
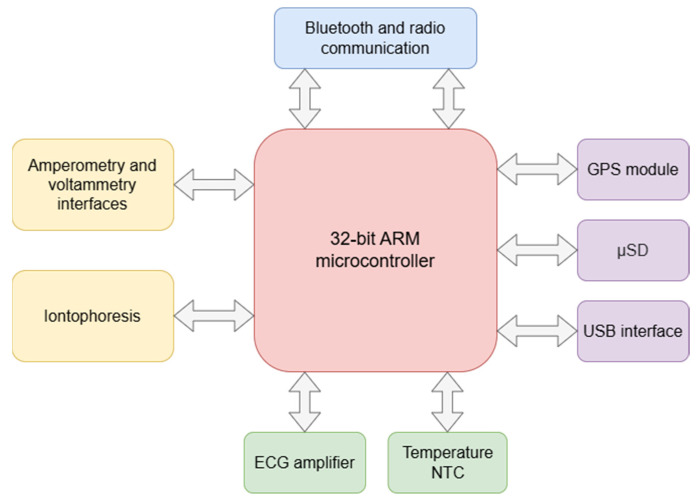
The block diagram of the BACQ device.

The wearable data acquisition device, BACQ, was designed as a central unit capable of reading data from various sensors, as well as transferring the data, performing simple processing algorithms and controlling the BetaStim stimulator. BACQ is based on the STM32H743VIT6 microcontroller by ST Microelectronics (Plan-les-Ouates, Switzerland), as shown in Figure 2. The device includes interfaces which enable measuring multiple physiological signals. It can track ECG (HR), body temperature and gather data from up to 6 electrochemical sensors. A single lead ECG was integrated for measuring HR. Body temperature is measured with a precise 10 kΩ NTC thermistor connected between analogue ground and one of three voltage analogue inputs. The voltage across the NTC thermistor is induced by a constant 100 µA current flow generated by an integrated precise current source. The interface for electrochemical sensors is based on the single channel electrochemical front-end circuit, AD5941, by Analog Devices, Inc. (Wilmington, MA, USA), and six-channel multiplexer which enables data acquisition from 6 separate electrochemical sensors. The proposed circuit can perform the common electrochemical techniques chronoamperometry (CA) and cyclic voltammetry (CV) with a voltage and current range of 0.2–2.1 V and 50 pA–3 mA respectively, as well as single point electrochemical impedance measurements (EIS) at different frequencies ranging from 100 Hz to 10 kHz.

Additionally, an iontophoretic stimulator was also integrated in BACQ, which allows the application of pulsed low currents (<0.5 mA/cm^2^). This module is intended for non-invasive biomarkers extraction through transdermal reverse iontophoresis. By this means, both the charged and uncharged polar (bio)molecules can be conducted across the skin towards the electrochemical sensor, accelerating the transmission of biomarkers into the bioanalytical layer.

Apart from measuring physiological and biomedical signals, the device was also able to keep track of its current geographic location as it integrated the GNSS module L86-M33 manufactured by Quectel Wireless Solutions Co., Ltd. (Shanghai, China).

The device also included a slot for a µSD memory card allowing it to log all the acquired data. This feature is particularly useful in case of communication failure. In such situations, the acquired data is stored on the memory card, preventing its loss. Upon restoring the communication, all the logged data is automatically transmitted to the corresponding recipient.

#### 2.1.3. Stimulation Device

The stimulation device, BetaStim, was specifically designed to perform electrotactile stimulation as well as to support the generation of stimulation patterns such as the ones presented in [35]. Electrotactile stimulation was chosen as a medium for providing feedback to a first responder rather than visual or audio signals as tactile sensation was deemed both less obtrusive and more efficient than the latter two [32,33,44].

**Figure 3:**
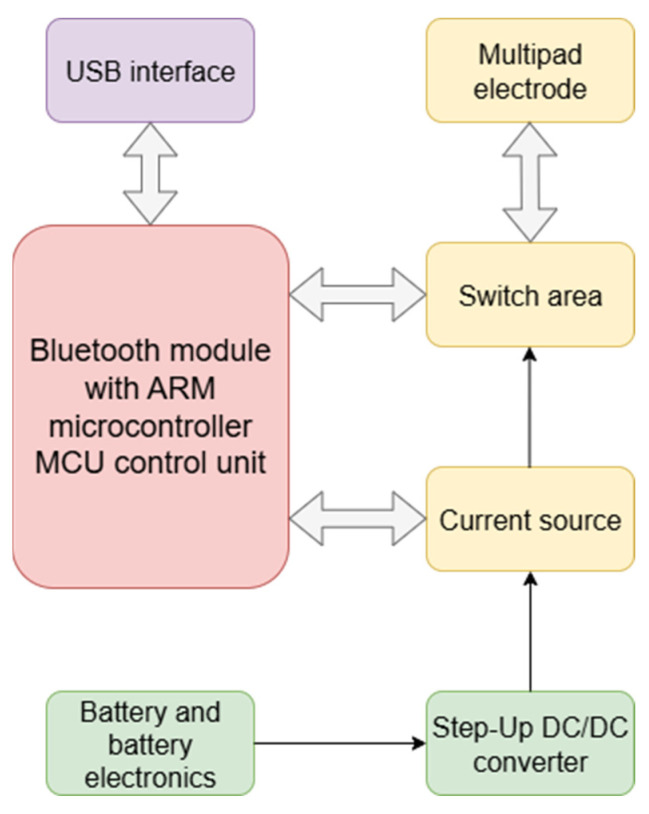
The block diagram of the BetaStim device.

The wearable BetaStim device structure, shown in Figure 3, encompasses several subsystems responsible for generating current pulses and enabling stimulation. In order to provide high enough voltage to enable current flow through the relatively high electrode-skin interface impedance a step-up DC/DC converter was integrated into the device. This converter is capable of boosting the battery’s 3.7 V to a voltage level of up to 150 V. The biphasic current source was based on a current controlled H-bridge topology which is able to produce either a monophasic or biphasic current pulse. The amplitude of the current pulse is controlled by a control loop which is integrated into the H-bridge. The switch area allows multiplexing a signal from the H-bridge and driving it to one or more of the 16 output channels. The Silicon Labs, Inc. (Austin, TX, USA) Bluetooth Low Energy module with an integrated high-performance microcontroller was used as both the control unit and communication interface for the BetaStim device. This component was chosen as it met all the requirements in terms of controlling the subsystems responsible for performing stimulation and also contributed to the reduction in the dimensions of the device compared to other microcontrollers, which is critical for wearable devices.

### 2.2. Feedback Architecture

The developed system supports two distinct modes for providing feedback to the wearer: a remote feedback loop and a local feedback loop. These modes differ significantly in terms of algorithmic complexity, system autonomy, and communication dependency. This dual-loop architecture ensures both high-level data interpretation and uninterrupted operation under degraded communication conditions.

#### 2.2.1. Remote Feedback Loop

The remote feedback loop is intended to rely on complex and computationally demanding algorithms executed at the command centre, primarily for physiological data analysis and decision-making during first responder operations. The execution of advanced physiological analytics and decision-making algorithms is a separate developmental stage and is not implemented in the present work. This study is limited to establishing the data pipeline, wherein physiological information acquired by the BACQ device is transmitted to a remote hub to enable, but not yet perform, more complex algorithmic analysis.

To enable this mode, intra-team communication is established using a custom-built RF link operating at 868 MHz, independent of existing telecommunication infrastructures such as GSM. Each team member’s BACQ device transmits data to the team leader’s BACQ device over this RF link. The team leader’s device then forwards the aggregated data to the MOTOTRBO Ion smart portable radio via Bluetooth. Subsequently, the data is transmitted to the remote command centre. Based on the received information, appropriate commands are sent back through the same path, eventually reaching each responder’s BetaStim device, which executes electrotactile stimulation to provide feedback.

A remote feedback loop theoretically enables the use of detailed physiological modelling and machine learning-based stress classification that are too computationally intensive for local execution. However, this study does not implement these advanced algorithms, focusing instead on the underlying communication infrastructure. Importantly, the functionality of this approach is critically dependent on the integrity of the communication channels. In the event of a communication failure, e.g., loss of RF link or Bluetooth connectivity, this feedback pathway becomes non-operational.

#### 2.2.2. Local Feedback Loop

To ensure system robustness and continuity of feedback during communication failures, a local feedback loop has been implemented. This mode supports the execution of lightweight, real-time algorithms directly on the BACQ device. It is designed to detect critical physiological thresholds and issue immediate feedback to the user without relying on any external communication or infrastructure.

In the local loop, physiological signals are acquired using the sensors integrated into the BACQ system (see Section 2.1.2). These signals are processed locally, and based on the outcome of this analysis, commands are issued to the BetaStim device via Bluetooth. BetaStim then generates electrotactile stimulation as feedback to the first responder. This architecture enables a fast and reliable response in high-risk scenarios where uninterrupted operation is essential.

Although the local loop cannot match the analytical depth of the remote system, it provides a fail-safe mechanism that ensures minimal viable feedback is always available, especially in mission-critical contexts.

## 3. Results of Experimental Verification

### 3.1. Performance Test of the Electrochemical Front-End

For the purposes of testing the electrochemical acquisition system, a custom LabVIEW application (National Instruments, Austin, TX, USA) was developed. The application allowed for the selection of the desired mode (iontophoresis, CA, CV, or impedance measurements) and the adjustment of relevant parameters corresponding to the chosen mode.

For CA, the adjustable parameters included the sensor bias [mV], time [min] and channel (1–6). Real-time data, the current measured in µA, is displayed, and the last value within the chosen time interval appears in the relevant window. New sample values are updated every second. For CV, the user sets the start voltage [mV], peak voltage [mV], duration [s], and channel (1–6). The chosen electrode is visually represented in an on-screen drawing. The scan rate [mV/s] is automatically calculated. After each cycle, data is plotted on a chart alongside previous data and stored. The number of completed cycles is also displayed. Finally, for impedance measurements, the user can specify the time [min] and channel (1–6). Each second, the returned RzMag [Ω] and Phase [°] values are displayed for three frequencies: 100 Hz, 1 kHz and 10 kHz.

**Figure 4:**
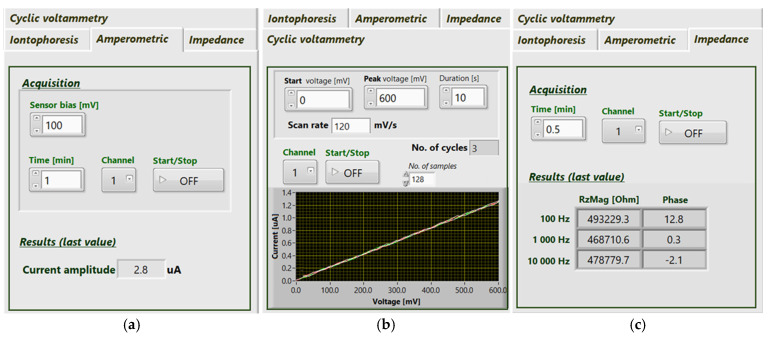
LabVIEW application for the configuration and monitoring of electrochemical acquisition data from the BACQ device: (**a**) amperometry, (**b**) cyclic voltammetry and (**c**) impedance measurement.

The electrochemical front-end was tested using reference electrical circuits to assess the accuracy of the electrochemical cell measurement. As an example of the test application, a 470 kΩ resistor was used to test the three acquisition modes and the results obtained are presented in Figure 4. The resistance that the device measures through the imposed resistor was calculated using Ohm’s law and was consistent with the nominal value of the reference resistor. These results confirm that the electrochemical module and its key functionalities operate correctly for the intended application.

The BACQ device’s electrochemical acquisition system was further tested using solutions of known properties. In-house-developed screen-printed carbon electrodes (named Kapsense) consisting of a three-electrode cell configuration were used. The electrodes were screen-printed onto a flexible polyethylene terephthalate substrate (ELECROM STS H.02-H.02–STD 125 µm) using screen-printable Henkel AG & Co. KGaA (Düsseldorf, Germany) inks for biomedical applications. Before evaluating the BACQ device, the electrochemical behaviour of the Kapsense electrode was characterized and validated. This was done with a standard ferri/ferrocyanide ([Fe(CN)_6_]^3−/4−^) redox probe which was chosen due to its well-known, fast, and reversible mono-electronic electron transfer properties [45].

### 3.2. Evaluating the BACQ Electrochemical Front-End for Dehydration State Measurement

The verification process of the electrochemical front-end was conducted as a benchtop feasibility study to evaluate the functionality of the electrochemical acquisition system under controlled laboratory conditions. The aim was to validate core measurement capabilities prior to further development and field testing. In addition, custom-made electrodes were manufactured to enable comprehensive evaluation. It should be noted that, for deployment in real-world applications, comprehensive calibration of both the acquisition system and the electrodes is required. However, this calibration procedure is beyond the scope of the present work and will be addressed in a future study.

To evaluate the application of the BACQ system, the EIS technique was selected over CV and CA. EIS offers a distinct advantage by enabling the characterization of complex electrochemical processes at the skin–sensor interface across a wide frequency range [46]. While CV and CA are valuable for probing redox reactions and transient current responses, bulk ionic strength and electrical conductivity are closely linked to hydration status. Importantly, the current implementation of EIS in the BACQ system is not intended to provide selective detection of Na^+^ ions, but rather to serve as a non-selective proxy for total ionic conductivity, reflecting overall electrolyte concentration in sweat.

To assess the performance of EIS within the BACQ device, artificial sweat solutions spiked with different concentrations of NaCl (1–250 mM), covering the physiological range, were used to simulate changes in sweat conductivity. These solutions were measured using both the BACQ system and the commercial potentiostat Autolab PGSTAT 204 (Metrohm Autolab B.V., Utrecht, The Netherlands) at identical frequencies. For each measurement, 60 μL of solution was applied on the screen-printed electrode. The experimental data were interpreted by analyzing the absolute impedance as a function of frequency, Z(f), and NaCl concentration. The resulting impedance response was used to derive a conductivity-based hydration index, which reflects changes in bulk ionic content rather than Na^+^-specific concentration.

**Figure 5:**
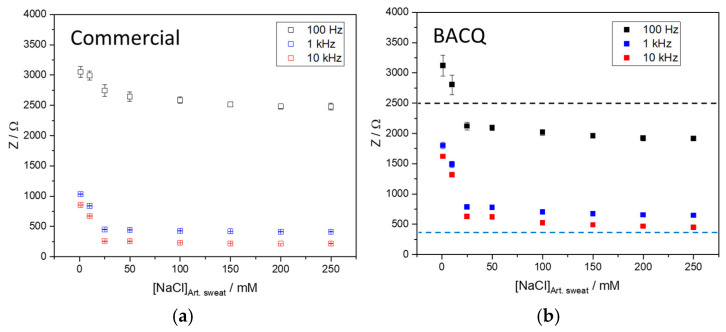
Impedance response of different spiked NaCl artificial sweat solutions measured with (**a**) commercial instrument and (**b**) BACQ at frequencies of 100 Hz, 1 kHz and 10 kHz.

Figure 5 presents the impedance magnitude (Z, RzMag) trends across frequencies for each salt concentration, as measured by both devices. As expected, impedance values decreased with increasing salt concentration and thus conductivity of solution at all frequencies in both systems, following an approximately exponential relationship. As illustrated, the same impedance-[NaCl] trend is seen, demonstrating the capacity of the BACQ device to monitor impedance changes in solution due to varying ionic content. The coefficient of variation for *n* = 3 was between 0.4% and 3%, demonstrating the reliability of the BACQ system.

Both devices exhibit consistent impedance trends across the tested frequencies and concentrations, which is critical for this evaluation and confirms the reliability and suitability of the BACQ device for its intended application. Although differences in absolute impedance values are observed at both low and high frequencies, these discrepancies do not compromise the device’s usefulness, as the assessment of hydration status relies primarily on relative trends rather than exact magnitude matching. The observed offsets are attributable to additional impedance and filtering effects introduced by internal circuitry, including protective components, input filtering, and differences in analogue front-end design. Furthermore, calibration of the electrochemical acquisition chain, encompassing both the BACQ device and the Kapsense electrodes, could further reduce the observed offsets and improve agreement with the commercial reference.

The frequency range of the applied AC signal significantly influences the interpretation of EIS results and affects the sensor’s sensitivity to bulk solution impedance quantification. Based on the findings, 1 kHz and 10 kHz can be considered optimal measurement frequencies, as they minimize frequency-dependent deviations not arising from impedance changes. Furthermore, since sweat can vary considerably among individuals, both 1 kHz and 10 kHz frequencies present enough sensitivity to detect changes on conductivity through impedance measurement in this range.

### 3.3. Performance Tests of Heart Rate and Temperature Acquisition

Two test scenarios were conducted to evaluate the performance of the HR and temperature acquisition system. The first scenario was performed in laboratory conditions and involved exercises with dynamic movements, while the second scenario was conducted as a field trial and involved regular movements typical of first responder missions. In the laboratory scenario, 10 examinees performed a set of treadmill exercises with varying intensity levels. HR signals were recorded throughout the entire workout procedure while the functionality of the NTC thermistor interface for temperature acquisition was verified separately using precision resistors under laboratory conditions. For the purposes of this test, the sampling rate was set to 1 sample per second to match the sampling rate of the reference device and no additional signal processing was performed.

The second scenario involved a field trial conducted within the scope of the SIXTHSENSE project [2,47] to verify system performance in realistic operational conditions. Twenty first responders participated, including 8 mountain rescuers and 12 firefighters, 18 male and 2 female with an average age of 35.8 ± 12.3 years. Each participant signed an informed consent form describing the study aims and experimental procedure. The study was conducted in accordance with the Declaration of Helsinki and the experimental protocol was approved by the local ethics committee (number 1322/III-19, date 17 March 2021). Participants performed actions which simulate typical first responder missions during two sets of four five-minute exercises separated by one-minute pauses. During the field trial, the sampling rate was set to 10 samples per minute, and the data was processed with a low-pass filter.

### 3.4. Results of the Heart Rate and Temperature Acquisition Tests

**Figure 6:**
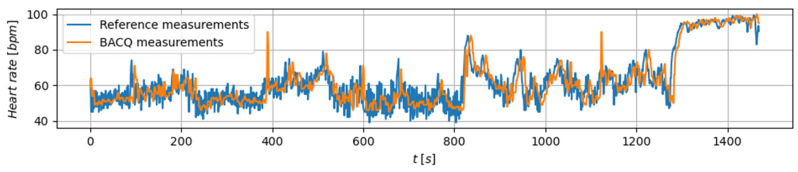
Heart rate measurements of an examinee during the treadmill workout. The diagram shows raw, unprocessed data where moving artifact is present and clearly visible.

The results of HR acquisition for one examinee in the first test scenario are shown in Figure 6. The figure compares measurements obtained using the BACQ device with those from a commercial device. Due to the dynamic nature of the movements, the recorded signals contain expected motion artifacts, which are noticeable in both the measurements obtained by the commercial device and the BACQ device.

**Figure 7:**
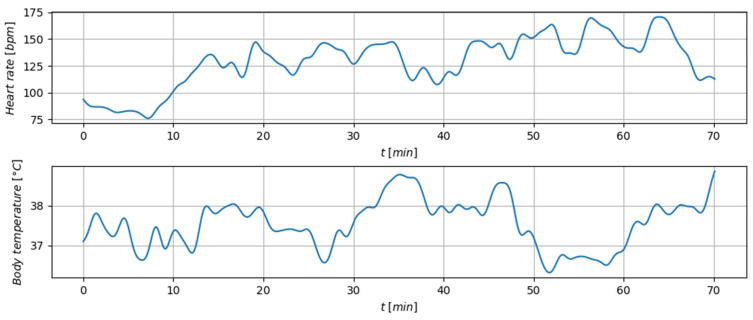
The calculated heart rate (beats per minute) and body temperature (°C) acquired during the field trial for one participant. The diagram shows the data processed using a low-pass filter.

Figure 7 shows an example of HR and body temperature recorded from one subject during the field trial across two exercise blocks. An increase in HR was noticeable at the beginning of each of the eight exercises, and a lower HR value at the beginning and end of the exercises, and during the longer pause between two exercise boxes (around the 40th min).

The physiological signals presented in Figure 6 and Figure 7 are shown as representative examples illustrating the capability of the system to acquire, transmit, and visualize physiological data during real operational exercises. The main objective of this work is to demonstrate the integrated sensing, telemetry, and feedback architecture under realistic field conditions. A comprehensive cohort-level statistical analysis across all participants (e.g., heart rate estimation accuracy against reference systems, motion-artifact quantification, and temperature accuracy) is outside the scope of the present study and will be reported in a separate study focused specifically on physiological signal processing and algorithm validation.

### 3.5. Communication Interface and Electrotactile Feedback Evaluation

During the field trial, both HR and body temperature were continuously transmitted to a remote command centre. Communication proved to be reliable, both between the devices worn by each member of a team and between the team leader’s device and the command centre. During the field trials reported in this study, the focus was on data collection to support algorithm development and no real-time stress estimates were generated. A study on this topic was published in [48].

The findings concerning electrotactile feedback were already published in an earlier paper [35]. The system allowed for the adjustment of stimulation amplitude, a crucial feature due to changes in perceived stimulus intensity resulting from participant exhaustion, electrode–skin contact variations, and electrode malfunctions. Recognition was tested in two sessions: pre-workout (verification) and post-workout. In the pre-workout session, participants received 360 messages in total (20 participants × 6 message patterns × 3 repetitions). The mean recognition accuracy was 93.3%, with ten participants achieving a perfect score and two participants scoring lowest at 72.2%. In contrast, during the field trial, several hardware-related issues typical for demanding operational environments occurred, such as electrode detachment and occasional connector disconnection during equipment donning and physical exercises. As a result, the number of delivered messages varied across participants. In total, participants reported receiving 558 messages from the command centre, resulting in an overall message recognition success rate (SR) of 73.5%, with individual SRs ranging from 52.4% to 87.2%. In summary, participants reported 918 messages, achieving a success rate of 81.3%. These results demonstrate high recognition accuracy under controlled conditions and more variable performance in the field, highlighting the impact of technical and physical factors on electrotactile feedback reliability.

To assess the impact of electrotactile stimulation on task performance, participants completed a post-trial questionnaire evaluating perceived distraction and difficulty during operational tasks. Among the 20 participants, 3 participants found the stimulation slightly distracting. Regarding task difficulty, 3 participants reported that performing their operational tasks felt somewhat more challenging during stimulation, while the remainder did not perceive any increase in difficulty.

## 4. Discussion

This paper presented the design, implementation, and validation of a wearable prototype system developed to assist first responders by continuously monitoring their physiological parameters and providing health-informed feedback through electrotactile stimulation. The system comprises three main components: the BACQ physiological signal acquisition unit, the BetaStim stimulation unit, and a powerful communication interface. Both devices were integrated into a wearable system and connected to a remote command centre for data storage and analysis and generating feedback messages.

Initial validation was conducted under controlled laboratory conditions. The BACQ device demonstrated the ability to generate and process electrochemical signals required for sweat analysis. A custom LabVIEW application enabled testing on an equivalent human model, verifying the system’s capability to acquire data from electrochemical sensors. EIS was employed to obtain indicators related to dehydration. The results obtained from EIS measurements confirm that the BACQ system performs reliably across a range of frequencies and electrolyte concentrations. Despite minor differences in impedance behaviour compared to a commercial potentiostat, the BACQ device demonstrates consistent and accurate electrochemical responses. These findings validate its suitability for monitoring hydration status through detection of impedance changes caused by sweat at both 1 kHz and 10 kHz, supporting its application in wearable and portable health monitoring technologies.

Heart rate measurements were consistent with the reference values, and although occasional artifacts were observed (e.g., around the 390th and 1120th s in Figure 6), they were easily filtered out and did not interfere significantly with signal processing. Similarly, temperature measurements conducted using precision resistors in the laboratory confirmed the system’s ability to track body temperature with sufficient accuracy. Field trials involving firefighters and mountain rescuers further evaluated the system’s performance in operational environments. The system successfully recorded and transmitted heart rate and temperature data to the command centre in real time. The electrotactile stimulation system achieved a message recognition success rate exceeding 80%, indicating strong reliability even in dynamic field conditions. The participant feedback indicates that the electrotactile stimulation was generally well tolerated, with most users reporting no distraction. These findings suggest that, while minor adjustments to electrode placement and system ergonomics may further improve user experience, the current implementation is practical and effective as a communication channel for first responders under operational conditions.

In addition to its integrated use, the system was designed with a modular architecture, allowing its components, the BACQ and BetaStim devices, to be used independently for other research or practical applications. This flexibility broadens the potential impact of the technology across multiple domains, including health monitoring, sports science, and rehabilitation.

Future work will focus on expanding the system’s capabilities. This includes testing additional electrochemical sensing techniques, such as chronoamperometry, under laboratory conditions; acquiring sweat-based data from human test subjects; and improving the effectiveness and success rate of the electrotactile feedback mechanism. The results presented here demonstrate the feasibility and promise of the proposed system as a robust platform for physiological monitoring and closed-loop feedback in high-stakes environments.

## Data Availability

The raw data supporting the conclusions of this article will be made available by the authors on request.

## Abbreviations

The following abbreviations are used in this manuscript:

HR	Heart rate
RF	Radio frequency
BACQ	Beta acquisition device
NTC	Negative temperature coefficient
CA	Chronoamperometry
CV	Cyclic voltammetry
GSM	Global system for mobile communications
EIS	Electrochemical impedance spectroscopy
RE	Reference electrode
WE	Working electrode
CE	Counter electrode
ECSA	Electrochemically active surface area
BPM	Beat per minute
SR	Success rate
